# Sustainable Innovation: Turning Waste into Soil Additives

**DOI:** 10.3390/ma16072900

**Published:** 2023-04-06

**Authors:** Daria Marczak, Krzysztof Lejcuś, Iwona Lejcuś, Jakub Misiewicz

**Affiliations:** 1 Institute of Environmental Engineering, Wrocław University of Environmental and Life Sciences, 50-363 Wrocław, Poland; 2 Institute of Meteorology and Water Management-National Research Institute, 01-673 Warszawa, Poland

**Keywords:** circular bioeconomy, sustainable technology, waste management, biodegradation, natural fibres

## Abstract

In recent years, a dynamic increase in environmental pollution with textile waste has been observed. Natural textile waste has great potential for environmental applications. This work identifies potential ways of sustainably managing natural textile waste, which is problematic waste from sheep farming or the cultivation of fibrous plants. On the basis of textile waste, an innovative technology was developed to support water saving and plant vegetation- biodegradable water-absorbing geocomposites (BioWAGs). The major objective of this study was to determine BioWAG effectiveness under field conditions. The paper analyses the effect of BioWAGs on the increments in fresh and dry matter, the development of the root system, and the relative water content (RWC) of selected grass species. The conducted research confirmed the high efficiency of the developed technology. The BioWAGs increased the fresh mass of grass shoots by 230-420% and the root system by 130-200% compared with the control group. The study proved that BioWAGs are a highly effective technology that supports plant vegetation and saves water. Thanks to the reuse of waste materials, the developed technology is compatible with the assumptions of the circular economy and the goals of sustainable development.

## 1. Introduction

The continuous modifications in the environment caused by human economic activity often lead to the irreversible deterioration of the milieu, posing potential human health and ecological risks [1,2,3,4,5]. The ecosystem is facing numerous types of environmental pressure, including, among others, climate change, water deficits, and soil degradation, along with the increasing need to provide food and water safety for the growing population [6,7,8,9]. As a result, water access and fertile soil access are indispensable elements of the sustainable development of today’s world and are at the same time very difficult to achieve and maintain for a long period. According to analyses, failure to introduce sustainable solutions in water management and soil protection will lead to severe threats for human health and environment quality [10,11,12,13,14]. This has resulted in a growing interest in various types of soil additives that improve the retention capacity and fertility of soils.

As far as water retention is concerned, popular soil additives include, among others, zeolite, attapulgite, and superabsorbents (SAPs), which effectively reduce the consumption of water for irrigation and mitigate the effects of droughts [15,16,17,18,19,20]. Regarding the improvement in soil fertility, a wide range of fertilizers that guarantee obtaining the appropriate quality of crops in a short time and improve production are available [21,22]. According to statistics, the consumption of chemical fertilizers was 198 million t in the world, including 100 million t of nitrogen fertilizers (2016) [23]. Unfortunately, they have some disadvantages, and above of all, they have a negative impact on the environment [24,25,26]. The main problem caused by the excessive long-term use of soil additives, especially in the context of artificial fertilizers, is the deterioration of soil fertility, and quality and amount of crops, and even soil degradation and loss of biodiversity [27,28,29,30,31]. Another type of soil additives that have become particularly popular in recent years in environmental and hydraulic engineering are geotextiles. They may perform mechanical, hydraulic, and biological functions if applied to engineering objects [32,33,34,35]. They work very well in agriculture and horticulture as retention-enhancing materials and natural fertilizers [36,37]. Unfortunately, although they have numerous advantages, they pose a significant burden for the environment, particularly because a vast majority of them is produced using synthetic fibres [38,39,40]. The textile industry generated almost 2 million tonnes of CO_2_ (2015) and consumed approximately 80 billion cubic meters of water, which indicate a pollution level that threatens the life of humans and animals [41,42,43,44]. At the same time, one should bear in mind that plastics stored in soil may release complex mixtures of chemical substances into the environment and that their hydrophobic surface allows them to adsorb and store high concentrations of organic pollutants and heavy metals [45]. Global plastic production reached 3.68 billion tons in 2019. It is estimated that the upward trend will continue until 2050; thus, the global production of plastics may increase to 25 billion tons [46]. Once released into the environment, these materials are prone to degrade, generating microplastics and nanoplastics, which can cause a broad range of toxicological effects on animals and humans [46,47,48]. Moreover, a rapid increase in the production of plastic waste, which is mainly due to the short period of plastic use, has been noted in the last decade [49]. According to estimations, the life of approximately 40% of plastic products does not exceed one month, and materials used in agriculture, horticulture, and environmental engineering are rarely used for a period longer than one or two vegetation seasons [37,50]. As a result, it is desirable to seek and apply alternative solutions that take into account the current environmental challenges.

Responsible production with the use of biodegradable materials is crucial in reducing the consumption of fossil fuels, greenhouse-gas emissions, and the storage of plastics in landfills [49]. The application of materials that undergo gradual biodegradation in soil offers a promising outlook [51,52,53,54]. The choice of biodegradable materials is particularly advisable in short-term solutions [55,56,57,58]. When applied to soil, they improve its mechanical properties, and as a result of biodegradation, they release compounds promoting vegetation [59,60,61,62]. In most cases, the choice of natural fibres such as wool, jute, or linen for manufacturing geotextiles is also reasonable from the economic point of view [63,64,65,66]. These materials are usually by-products of breeding sheep, cultivating fibre plants, or manufacturing textiles in the clothing industry. Currently, attempts at applying natural fibres in environmental engineering and agriculture, where they act as elements that support plant growth and provide anti-erosion protection to civil engineering structures, are being made [36,64,67]. This subject has not been thoroughly analysed yet; the available materials are not always effective, and the conducted research focuses on a narrow range of applications. As a result, there is clearly a need to use the potential of such materials and to develop technologies that meet the current needs of the market while taking into account the sustainable development goals and the objectives of circular economy, such as improving the efficiency of water management and soil fertility, reducing the amount of waste, and limiting soil degradation.

Biodegradable water-absorbing geocomposites (BioWAGs) have been developed to solve the problems discussed above. This technology has a three-dimensional form that retains water in the form of gel so that it is available for plants. A BioWAG consists of three main elements: biotextile, which separates the whole structure from the soil; a superabsorbent polymer, which absorbs water and its solutions; and an internal skeleton structure, which provides space for free water absorption [68,69]. The plant root system can freely grow into the WAG interior and take up the water stored in the SAP [70]. The geocomposite water storage process is very efficient and can take place many times [68]. WAGs can be manufactured both in a durable version (WAGs) based on synthetic materials and in a biodegradable version (BioWAGs). The use of geocomposites has a positive effect on the development of the plant root system, increasing the erosion resistance of engineering structures, such as flood embankments. This technology also reduces evaporation from the soil and improves the biometric parameters of plants [68,71,72,73,74].

This work illustrates the need for a sustainable approach to natural resources and indicates the possibility of using waste materials in a closed cycle. This paper presents a new, sustainable approach to the management of textile waste materials to reduce environmental pollution, recover valuable nutrients readily available to plants, and reduce the water consumption necessary for irrigation. We explored the possibilities of biodegradable waste management using innovative technology in the form of BioWAGs. The research presented was conducted under actual field conditions so as to take into consideration the influence of external factors. The major purpose of the work was to determine the potential of textile waste materials for the production of an innovative technology to support plant vegetation and save water in the form of BioWAGs and to determine BioWAG effectiveness under field conditions. We analysed the effect of BioWAGs on the increments in fresh and dry matter, the development of the root system, and the relative water content (RWC) of selected grass species.

## 2. Materials and Methods

### 2.1. Site Characteristics

The experiment was carried out at Agricultural and Hydrological Observatory Wrocław-Swojec (51°07′ N, 17°10′ E), Wroclaw University of Environmental and Life Sciences, Poland, located in the northeastern area of the city. The duration of the experiment was from May to October. Wrocław is located in SW Poland, in the moderate climate zone. The annual precipitation in Lower Silesia is between 560 and 600 mm [37,75].

### 2.2. Climatic Conditions

**Figure 1:**
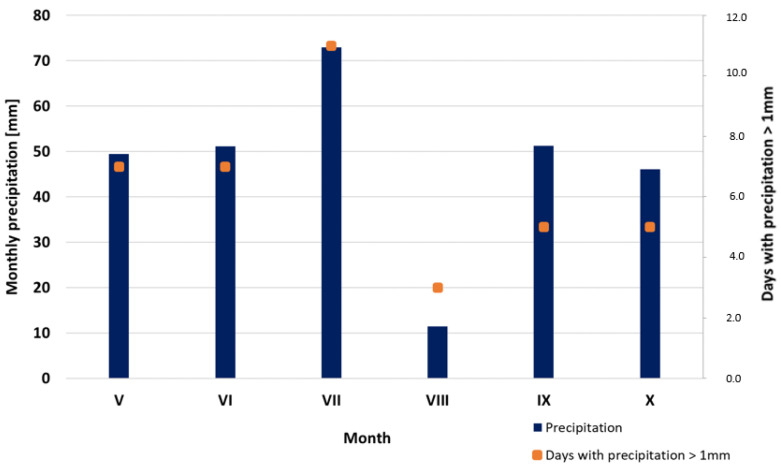
Histogram of total precipitation by month in the analysed period. Meteorological data were obtained from Faculty Agricultural and Hydrological Observatory Wrocław-Swojec (WOAiHW-S).

The average daily temperatures in the test period ranged from 5.1 to 25.9 °C (May–October 2018). Total monthly precipitation ranged from 11.4 mm in August to 72.9 mm in July (Figure 1). During the test period, 38 days with rainfall exceeding 1.0 mm were noted. The highest total monthly precipitation (72.9 mm) was observed in July, and the lowest one, in August (11.4 mm). During the test period, the average temperature was 16.6 °C, which exceeds the long-term average (1971–2000) by 1.7 °C. The total rainfall was 282.1 mm, which is 86.8 mm lower than the average annual rainfall (1971–2000) [37,76].

### 2.3. Materials

The tests were conducted with the use of prototype BioWAGs prepared in the form of mats with spatial dimensions of 0.22 × 0.22 × 0.22 m. The WAG prototypes consisted of Aquasorb 3005 KL superabsorbent polymer (SNF FLOERGER, Andrézieux, France); a wooden skeletal structure (0.20 × 0.20 × 0.02 m); and a biodegradable nonwoven, which constituted the sheath (Table 1). The used superabsorbent polymer is a cross-linked copolymer of acrylamides and potassium acrylate that undergoes gradual biodegradation under the influence of environmental factors, including selected soil bacteria [70]. Bacteria causing slow SAP degradation include *Variovorax boronicumulans*, *Enterococcus faecalis*, *Klebsiella pneumoniae*, and *Geobacillus thermoglucosidasius* [77,78]. All nonwovens used in the production of the WAGs were analysed based on their physical and mechanical properties before and after biodegradation (after one vegetation season). The surface masses of the scoured nonwovens were BA, 368 g∙m^−2^; BB, 287 g∙m^−2^; BC, 333 g∙m^−2^; BD, 289 g∙m^−2^; and BE, 308 g∙m^−2^. The detailed results concerning the parameters of nonwovens before and after biodegradation were published in the paper by Marczak et al. [37].

### 2.4. Preparation of BioWAG Prototypes and Test Sites

The prototypes of the BioWAGs were prepared using rectangular fragments of nonwovens (0.44 × 0.22 m), which were folded and sewn with cotton thread to create square mats of the dimensions of 0.22 × 0.22 m. A wooden skeletal structure and the SAP in the form of dry granules ranging from 0.50 mm to 2.00 mm were placed inside the mat, which was then sewn shut. The dose of the SAP was selected so that after swelling, the SAP could fill the free space inside the geocomposite. The WAGs prepared in such a way were soaked in water before application in soil and then placed at the bottom of a hole with the diameter of 0.30 m prepared on the test field. The walls of the hole were additionally protected with a synthetic sheath to ensure the same area for the development of the plant root system at all sites. The applied geocomposites were covered with a layer of humus (of the grain size distribution of loamy sand) with a thickness of approx. 0.15 m. The used soil was classified according to the United States Department of Agriculture (USDA) classification [79]. The study determined the basic parameters of the soil, such as pH 6.20 1 M∙dm^−^^3^ KCl, specific weight of 2.65 g∙cm^−^^3^, bulk density of 1.65 g∙cm^−^^3^, and soil organic matter (SOM) of 2.93%. Detailed soil parameters are presented in more detail in the article [69]. All sites were sown with 0.63 g of grass mixture seeds (which corresponds to 200 $\mathrm{kg}\cdot {\mathrm{ha}}^{-1}$) [37].

### 2.5. Description of the Experiment

**Figure 2:**
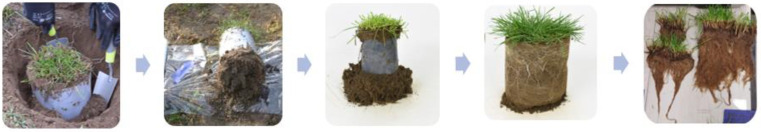
Preparation of the sample for laboratory tests.

The tests were conducted from May to October 2018. Each type of BioWAG and the control site were installed in 9 iterations. Samples were placed at random, so as to eliminate the influence of threshold conditions. All sites were regularly irrigated until grass sprouted and rooted (approx. 2–3 weeks after sowing). The same doses of irrigation were used at each site in order to ensure comparable vegetation conditions. After that time, no additional irrigation was conducted until the end of the vegetation season, so the amount of water stored in the BioWAGs depended on atmospheric conditions. The condition and growth of plants were regularly monitored by controlling fresh and dry weight, and the water balance of plants. In order to assess the root systems of plants, after the end of the vegetation season, roots, protected with plastic sheaths, were collected from the test sites (surface area: 0.0314 m^2^; height: 0.20 m). Samples were additionally protected with foil and transported to the laboratory in intact condition. After taking photographs for documentation and collecting the BioWAGs for further analyses, the root mass was soaked in water to remove soil particles (Figure 2). The cleaned root systems of grass were subjected to biometric analyses.

### 2.6. Research Methodology

#### 2.6.1. Measuring the Fresh and Dry Weight of Plants

Grass coverage at test sites was regularly monitored during the analysed period. Fresh weight and dry weight were measured regularly, every 7–8 weeks. During the analysed season, swaths were conducted 3 times, and measurements were taken in 9 iterations. All sites had the same surface area (0.0314 m^2^) defined by the plastic sheath installed in soil. Grass was manually cut, with shears, as close to the ground as possible. The cut growth was placed in a plastic bag and weighed after transporting to the laboratory. In order to obtain the dry weight, the grass was laid out in a laboratory drier with natural air circulation at the temperature of 70 °C and dried until a fixed weight of samples was obtained (48 h) [80].

#### 2.6.2. Relative Water Content (RWC) Measurement

The degree of plant hydration was assessed based on the relative water content (RWC). RWC was measured during each analysis of the dry and fresh weight of plants. Samples of grass blades collected from the field were placed in string bags and transported to the laboratory. In order to determine the RWC, we measured fresh weight (directly after collecting the grass blades), turgid weight (obtained after soaking the leaves in deionised water for 24 h), and dry weight (after drying in a laboratory dryer with natural air circulation, at the temperature of 70 °C, until a fixed sample weight was obtained). The RWC values were calculated using the following (Equation (1)) [80,81]:

(1) $$\mathrm{RWC}=\frac{\mathrm{FW}-\mathrm{DW}}{\mathrm{TW}-\mathrm{DW}}\cdot 100\;$$

where RWC is the relative water content (%), FW is the fresh weight (g), DW is the dry weight (g), and TW is the turgid weight (g).

#### 2.6.3. Analysis of the Development of Plant Root System

The root monoliths of plants were collected from the experimental field in November, after the growing season had passed. The measurements were taken in 9 iterations for each type of WAG and the control group. The condition and development of the root system were analysed with the use of biometric measurements. The roots were dried in a laboratory dryer with natural air circulation, at the temperature of 70 °C, and dried until a fixed weight of samples was obtained (48 h). Then, their dry weight was measured. The length and total density of the root system were also measured. The total density was calculated with the following (Equation (2)):

(2) $$\rho =\frac{m}{V}\;$$

where *ρ* is the density (g∙m^−3^), m is the dry weight (g), and V is the volume of soil from which the root system was collected (m^3^).

The monolith method was used to determine the depth distribution of the RLD [82,83]. The obtained measurements of root length and the known volume of soil were the basis for the determination of the root length density (RLD) index (3).

(3) $$\mathrm{RLD}=\frac{L_{T}}{V\;}\;$$

where RLD is the volume density of the root system (m∙m^−3^) L_T_ is the total length of the roots (m), and V is the volume of the soil from which the root system was collected (m^3^).

Then, the root system was divided into 0.05 m wide belts, and the dry weight of roots in specific layers and the designated density of roots in the 0.05 m thick layer were measured using the following (Equation (4)):

(4) $$\rho_{0.05m}=\frac{m_{0.05m}}{V_{0.05m}}\;$$

where $\rho_{0.05m}$ is the density determined in the 0.05 m thick layer (g∙m^−3^), $m_{0.05m}$ is the dry weight determined in the 0.05 m thick layer (g), and $V_{0.05m}$ is the volume of the soil determined in the 0.05 m thick layer (m^3^).

### 2.7. Data Analysis

The study was carried out in a randomized system so as to eliminate the possible impact of environmental factors. Boxplot analysis was used to observe the data range and identify any invalid data. Boxplots show the data distribution, range, minimum and maximum range values, lower quartile, upper quartile, and median [84,85]. Statistics for all the boxplots were prepared using Excel v 2019.

## 3. Results

### 3.1. Plant Growth

The tests revealed that BioWAGs had a positive influence on all the analysed biometric properties of plants. Several weeks after sowing, the sites were covered with grass. The grass growing in sites where BioWAGs had been applied was in a much better condition than that in the control sites. The plants had an intense green colour and were higher, and the grass cover was much denser and more cohesive.

**Figure 3:**
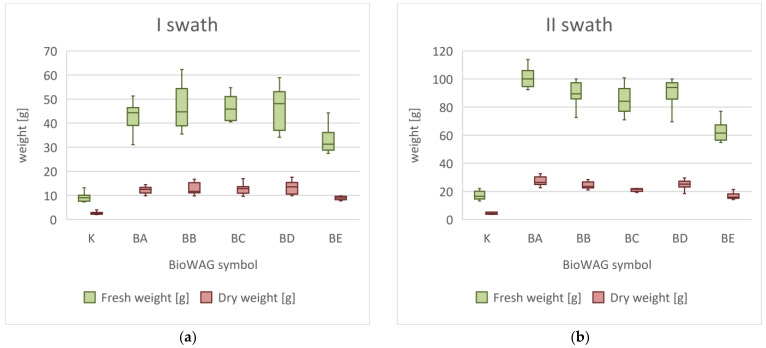

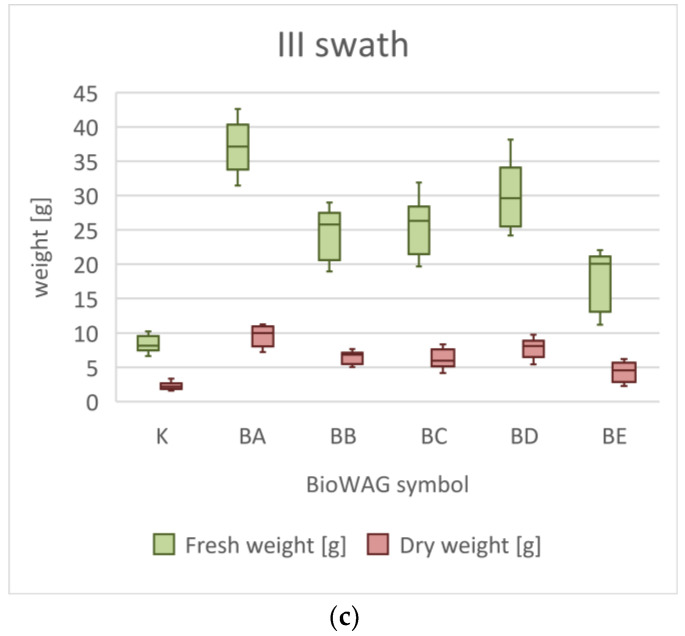
Influence of BioWAGs on the growth of above-ground biomass and dry above-ground mass. (**a**) Growth of fresh and dry grass after the first swath, (**b**) growth of fresh and dry grass after the second swath, and (**c**) growth of fresh and dry grass after the third swath. The diagrams show the first and third quartiles, medians, and minimum and maximum values.

Throughout the vegetation season, the increase in the fresh and dry weight of grass that was noted in sites with BioWAGs was higher than that in control sites (Figure 3). As early as after the first swath, the increase in the fresh weight of grass from sites with WAGs was, on the average, from 264 to 410% higher. Regarding the dry weight, the increase was 241 to 377% higher. At the same time, the highest effectiveness for both the fresh and dry weight of plants was noted in sites marked with the BD symbol. The highest effectiveness of WAGs in the whole season was noted after the second swath. The increase in the fresh weight of grass from test sites was 267 to 490% higher, and the increase in dry weight, from 248 to 467% higher. This time, the best results were observed in sites marked with the BA symbol, which resulted from the intensified biodegradation process of the material and the releasing of N and K into the soil. The increase after the third swath was 110 to 344% higher for fresh weight and 95 to 325% higher for dry weight. The best effects were noted, once again, in the sites marked with the BA symbol.

The highest effectiveness of BioWAGs noted after the second swath was directly linked to the atmospheric conditions in the period preceding the swath. August was the month with the lowest total precipitation and the highest average temperature in the whole analysed period. At that time, the plants had already a well-developed root system, which, thanks to hydrotropism, was able to quickly reach the water retained in the geocomposite. After the plants had grown through it, single granules of the SAP were distributed along the roots, providing the plant with continuous access to water. Depending on the material used, the biodegradation process of the elements of the BioWAGs started or intensified. Plants in sites where BioWAG was applied were provided with a sufficient amount of water and nutrients in that period, regardless of the environmental and climate conditions, which undoubtedly contributed to intensified growth.

### 3.2. Root Growth

**Figure 4:**
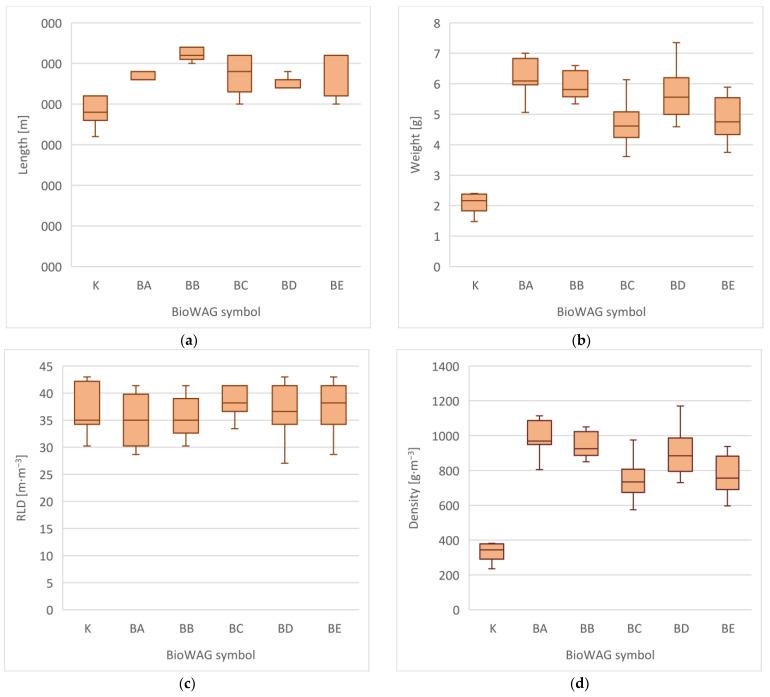
Biometric measurements of grass root system noted after the vegetation season (2018), depending on the type of BioWAG used: (**a**) length of the root system; (**b**) total weight of the root system; (**c**) values of the RLD index measured in reference to the total length of the root system; (**d**) total density of the root system. The diagrams show the first and third quartiles, medians, and minimum and maximum values.

The root systems of grass in sites with BioWAGs were dense and resistant structures that grew through the whole space of the geocomposite. In order to determine the efficiency of the WAGs at the end of the growing season, selected parameters of the root system were analysed, i.e., total length, total weight, weight at various depths, the RLD index, and density (total density and density at various depths). The analysis of these parameters revealed the favourable influence of the WAGs on the increase in and development of the root system (Figure 4). The application of BioWAGs caused increases in the length and in the RLD index of 24% (BA), 36% (BB), 25% (BC), 18% (BD), and 26% (BE) in comparison with the control group. The smallest differences were observed in BD sites and resulted from the low degree of biodegradation of this geocomposite. The remaining sites were subjected to more intense biodegradation, which created the possibility to collect them in an intact state from deeper levels. A detailed description of the progress in the biodegradation of the BioWAGs used in the research was presented in the paper by Marczak et al. [37].

Similarly, the dry weight and density of the root system were higher in sites where the BioWAGs had been applied. The application of this technology resulted in increases in biomass and root density of 202% (BA), 194% (BB), 125% (BC), 171% (BD), and 134% (BE) in comparison with the control block. In these cases, the best results were obtained in sites that were subjected to intensive biodegradation, at the same time improving the composition and fertility of the soil.

The analysis of the development of the grass root system in 5 cm thick layers allowed us to determine that the application of WAGs fostered creating a greater volume of roots in all the analysed depth ranges than in the control group. The root system was best developed in the 0.00–0.05 m range, where its density was 171% (BC) to 264% (BA) higher. In the 0.05–0.10 m and 0.10–0.15 m ranges, this parameter was 70% (BC) to 128% (BA) higher. The largest differences were observed in the 0.20–0.25 m range, where the root system density was 238% (BE) to even 688% higher (BA). Such large differences resulted from the lower reach of roots in control sites.

### 3.3. Relative Water Content (RWC) Measurement

**Figure 5:**
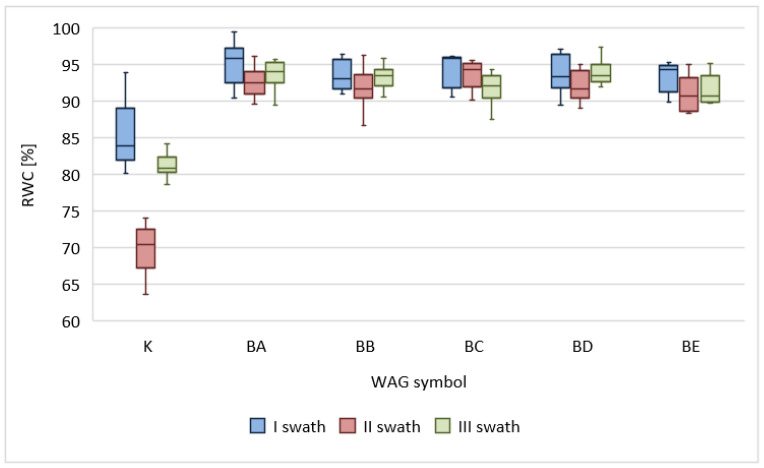
The influence of WAGs on relative water content (RWC). The diagram shows the first and third quartiles, medians, and minimum and maximum values.

The conducted analyses revealed a positive influence of the BioWAGs on the relative water content in grass leaves. During the whole vegetation season, the RWC in the test sites was approx. 10% to 35% higher than that in control sites (Figure 5). The best results were noted in samples collected after the second swath, which was directly linked to the disadvantageous atmospheric conditions in the period preceding the swath. August was the month with the lowest total precipitation and the highest average air temperature. As a result, plants in control sites were exposed to water stress. Regarding sites with BioWAGs, the RWC parameter did not fall below 91%, regardless of the type of applied water-absorbing geocomposite and the atmospheric conditions.

## 4. Discussion

The article presents the results of our research on the impact of biodegradable water-absorbing geocomposites on plant vegetation. The combination of the sorption capacity of the superabsorbent polymer and the biodegradable materials that are rich in nutrients allowed us to develop an innovative technology to save water and support plant growth. The results here presented demonstrate that this solution is highly efficient. The application of BioWAGs guarantees continuous access to water regardless of the atmospheric conditions. Moreover, it increases the growth of plants, mitigates the effects of water stress, and as a result, limits fertilizer use and reduces the negative environmental impact.

### 4.1. Relative Water Content (RWC)

Some of the effects of water deficit include diminished crops and limited development of plants. Numerous authors have confirmed that even mild stress caused by drought may lead to significant modifications in the physiological and chemical processes of plants. The yield of grass is closely linked to soil moisture content, i.e., grass responds to increased water content in the soil by significantly increasing the yield [86]. The results presented in this study confirm this phenomenon. The sites with BioWAG application provided plants with continuous access to water, which increased the crops by 270 up to 490% and reduced the stress caused by water deficit. The highest differences were noted under unfavourable weather conditions, when the plants were exposed to water stress. During that time, a noticeable decrease in growth in the control sites was noted. The literature reveals that droughts in Poland may cause a decrease in the yield of grass of approximately 30% [87]. Research by Staniak [88] indicated that the stress caused by water deficit led to a reduction in the dry weight of grass of approx. 31% in comparison with sites with optimum water content. In comparison with traditional irrigation, the application of BioWAGs ensures multiple times higher effectiveness, while at the same time, it makes it possible to save water and protect the environment. The optimum hydration of plants was also confirmed by higher values of the RWC index (91–95%), which were noted throughout the season in sites with BioWAGs in comparison with the control sites (69–85%).

### 4.2. Plant Growth

In recent years, superabsorbent polymers (SAPs) have been successfully used as soil additives, providing a reserve of water and of certain nutrients. These materials are an efficient tool for reducing the results of water stress, and they have a positive influence on plants cultivated in agriculture, horticulture, and environmental engineering [16,89,90]. However, in field applications, they have certain disadvantages that significantly limit their effectiveness. The main disadvantage is the reduced absorption capacity of SAPs under loading [79]. As far as WAGs are concerned, this weakness has been eliminated thanks to introducing the internal skeletal structure that ensures the free space required by the SAPs to properly swell. Lejcuś et al. [68] analysed the effectiveness of synthetic water-absorbing geocomposites in field applications. The non-biodegradable version of geocomposites had a positive influence on the biometric parameters of the turf overgrowing the slopes of the test embankment. The application of geocomposites resulted in an increase in the root volume of 130% and an in increase in their length of 29%. After the first year of the experiment, the dry weight of the above-ground parts of plants increased by 50%, and the fresh weight, by 58%, in comparison with the control group. The comparison of these results and the new, biodegradable version of WAGs allows us to state that the application of biodegradable materials improves the efficiency of this technology by even 6.5 times, depending on the analysed parameter and the material applied.

Water access and nutrient access are the main determinants of plant growth. Irrigation increases nitrogen availability, while the presence of nitrogen in the soil promotes water use, thus contributing to the growth of plants [91]. The unreasonable use of fertilizers, in particular nitrogen, has been the main threat for many years, both in terms of plant quality and environmental protection [92,93]. Users are continuously searching for alternative solutions, such as biodegradable materials or other natural fertilizers. Such materials include, among others, sheep’s wool, whose exceptionally rich composition makes it unique. It contains, among others, nitrogen, phosphorus, and potassium, which are available for plants in an easily accessible form [94,95]. In some of their studies, Broda et al. [96,97,98] noted a positive influence of waste wool applied to slopes on grass. In the places of application, the grass was much higher; it had a bright green colour and formed a dense, cohesive cover, as opposed to the control sites. Due to the degradation of wool, nitrogen was delivered to the soil, which fostered vegetation. Zheljazkov et al. [99] demonstrated that the process of biodegradation of wool was relatively slow, so it acted similarly to a slow-release fertilizer. The application of wool in a field experiment led to a two–three-fold increase in crops of *Digitalis purpurea* L. in comparison with the control group. Lal et al. also noted an improvement in soil fertility after the application of waste wool, as well as increased contents of organic carbon, nitrogen, and soil enzymes, of approx. 30% in comparison with the control group. The present study confirms the observations of other authors concerning the positive influence of wool on vegetation. In sites BA, BB, BC, and BD, where the percentage of wool was the highest, the growth of the above-ground parts of plants was 50–90% higher than in the BE site, which had the lowest content of wool. The FTIR analysis of the wool samples, which was described in detail in the paper by Marczak et al. [37], established that the biodegradation of keratin protein is accompanied by the release of nutrients that are easily accessible for plants into the soil. A correlation between the degree of biodegradation of the nonwovens and the development of the plant root systems at the depth below 0.20 m was also observed. In sites prepared with the use of needle-punched nonwovens (BB, BC, and BE), the degradation was so advanced that the textiles lost their integrity, and the root system was so well developed that it created a dense, cohesive structure and grew much deeper. This is particularly important when applying BioWAGs to engineering objects, such as flood embankments or road embankment slopes. Plants that grow roots in the layer of humus that is subjected to sliding grow through it, forming a mechanical connection with high resistance to the tearing of roots. The root system reinforces the soil, thus preventing landslides. Although numerous studies on the influence of soil additives on plant vegetation have been conducted, it is rather difficult to provide a straight comparison of the results presented here due to the innovative nature of the described technology. BioWAGs combine the properties of soil additives that improve retention, such as SAPs or zeolite, and fertilizers. Table 2 contains the results of research presented by other authors taking into account the influence on vegetation in terms of both increasing water retention and providing nutrients.

Based on the referenced data from the subject literature, noticeably better results were obtained with the application of BioWAGs. The technology described in this paper is not only characterised by retention capacity, but thanks to the presence of biodegradable materials, it also acts as a slow-release fertilizer. Regarding the analysed additives that increased retention, the growth of the above-ground parts of plants was high, i.e., from approx. 30% (zeolite) to 300% (0.4% hydrogel). On the other hand, applying fertilizers increased this parameter by 5% (270 kg N∙ha^−^^1^) to 47% (sheep manure). At the same time, the effectiveness of biodegradable WAGs was nearly 500% higher throughout the vegetation season, regardless of atmospheric conditions. The application of waste materials makes it possible to reduce environmental pollution, and it perfectly fits the principles of sustainable development and circular economy. The combination of superabsorbents and biotextiles, which have been very well known in agriculture, horticulture, and environmental engineering, is a competitive alternative to existing solutions, especially in regions exposed to droughts, degraded areas, and soils with low contents of plant nutrients.

## 5. Conclusions

Reasonable water consumption, excessive use of fertilizers, and reduction in soil pollution and waste generation are some of the main factors that influence the quality of the environment. So, to confront the current challenges, biodegradable water-absorbing geocomposites were developed as an innovative technology to support water saving and plant vegetation. The article provides an assessment of the potential of BioWAGs in field applications and their influence on plants. The results presented and described in the article allowed us to draw the following conclusions:

- BioWAGs are a highly efficient solution. The application of this sustainable technology guarantees continuous access to water regardless of the atmospheric conditions. Moreover, they increase the fresh and dry weight of plants, mitigate the effects of water stress, and as a result, make it possible to limit fertilizer use and reduce the negative environmental impact.
- Textiles used in BioWAGs can be successfully produced using the widely available fibres of animal and plant origin, such as linen, jute, or wool. As a result of biodegradation, geotextiles based on natural fibres gradually release, into the soil, easily accessible compounds that become natural fertilizers for plants.
- The research results indicate that BioWAGs have a positive effect on the development of above-ground and underground parts of selected grass species. Irrespective of the kind of biotextile applied, BioWAGs increased the fresh weight of grass shoots by 230–420% and the dry weight of roots by 130–200% in comparison with the control group.
- BioWAGs can reduce the effects of water stress, which was confirmed by the RWC results. The optimum hydration of plants was confirmed by the higher values of the RWC index (91–95%) that were noted throughout the season in sites with WAGs.
- The time of effective operation of BioWAGs may be adjusted to the requirements of plants and users’ expectations by using textiles with a particular time of biodegradation. All the materials applied in this work showed potential for at least one vegetation season. This time is suitable for the germination and development of plants used to protect slopes (grass and shrubs), ornamental plants, or agricultural crops.

## Figures and Tables

**Table 1:** Attributes of the analysed materials.

Sample Name	Composition of Raw Materials and Manufacturing Technology	Sample Photo
BA	99.4% washed wool and 0.6% synthetic seams; seamed textile	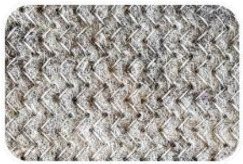
BB	100% washed wool; needle-punched nonwoven	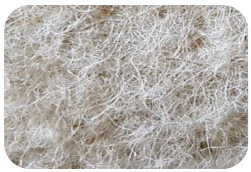
BC	90% washed wools and 10% jute; needle-punched nonwoven	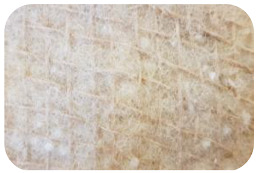
BD	90% washed wool and 10% jute; seamed textile	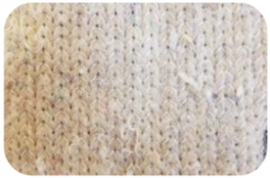
BE	50% washed wool and 50% linen; needle-punched nonwoven	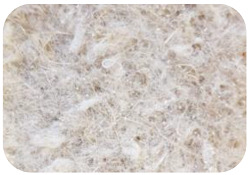

**Table 2:** Comparison of the influence of soil additives on plant vegetation.

Soil Additive	Plant	Brief Characteristics	Influence on Vegetation	References
- Hydrogel at various concentrations (0%, 0.2%, and 0.4%).	Agrostis stolonifera grass	- Controlled conditions (greenhouse). - Duration: 10 weeks. - Irrigation to ensure full saturation of the substrate with water.	- Increase in the fresh weight of shoots of 60–300% (higher with 0.4% hydrogel). - Increase in the fresh weight of root system of 30–120% (higher with 0.4% hydrogel).	[100]
- a-CSA-Hydrogel, and volcanic rock flour and bentonite. - b-CSA-Hydrogel, and digested fermentation compost and humic acids.	Cat grass (*Dactylis glomerata* L.)	- Field experiment (open-pit mine in Germany). - Duration: 2 years. - No irrigation.	- After 1 year: increase in the fresh weight of shoots of 90% (a-CSA); no differences were noted in the b-CSA site. - After year 1, no influence of additives on the growth of root system was noted. - After year 2, no influence of additives on the growth of above-ground parts was noted. - After year 2: increase in the dry weight of root system of 65% (a-CSA); no differences were noted in the b-CSA site.	[101]
- Sheep manure. - Avikhad. - Dirty waste wool (directly after shearing sheep). - Cleaned waste wool.	Barley	- Field experiment (semi-arid region of Delhi, India). - Duration: 2 years. - No irrigation.	- Increases in the fresh weight of shoots of 12% (waste wool), 16% (wool manure), and 47% (sheep manure). - Increase in crops of 50% (waste wool).	[95]
- Mineral: 100 kg N∙ha^−1^ and 30 kg K∙ha^−1^. - Digested: 26.3–30.3 t∙ha^−1^ biogas station (mineral N, organic N, P, K, Ca, and Mg).	Tall wheatgrass (Elymus elongatus subsp. ponticus) and reed canary grass (*Phalaris arundinacea* L.)	- Field experiment (Czech Republic). - Duration: 6 years. - No irrigation.	- Increases in the dry weight of tall wheatgrass of 14% and in that of reed canary of 6% (digested). - No significant influence of mineral fertilizer on the increase in dry weight was noted.	[102]
- Various doses of nitrogen fertilizer (330 kg N∙ha^−1^, 270 kg N∙ha^−1^, and 210 kg N∙ha^−1^).	Winter wheat	- Field experiment (North China Plain). - Duration: 3 years. - A total of 1 or 2 doses of irrigation per season.	- The highest increase in dry weight, 5–10%, was noted with the fertilizer dose of 270 kg N/ha.	[103]
- Zeolite (OZ). - Zeolite enriched with K (K-EZ).	Perennial ryegrass (*Lolium perenne* L.)	- Controlled conditions (greenhouse). - Duration: 3 months. - Regular irrigation.	- Increases in the dry weight of shoots of 10% (OZ) and 31% (K-EZ). - Increases in the dry weight of root system of 27% (OZ) and 68% (K-EZ). - No significant influence of additives on RWC was found.	[104]

## Data Availability

Not applicable.
